# A Review of Interventions for Drowning Prevention Among Adults

**DOI:** 10.1007/s10900-023-01189-6

**Published:** 2023-01-19

**Authors:** Justine E Leavy, Corie Gray, Malena Della Bona, Nicola D’Orazio, Gemma Crawford

**Affiliations:** Collaboration for Evidence, Research and Impact in Public Health, School of Population Health, Curtin University, Perth, WA Australia

**Keywords:** Drowning, Drowning prevention, Adults, Review, Evaluation, Evaluation design

## Abstract

Adult drowning is a complex and multifactorial public health challenge requiring community, national and global efforts to mitigate impacts. This study updates the evidence base for public health interventions that address adult fatal and non-fatal drowning. A systematic review was undertaken of the peer-reviewed literature for English-language primary studies published between 2011 and 2021describing a drowning intervention with adults. Twenty-two studies were included. Most studies (n = 16) were conducted in high-income countries. Yearly trends in drowning prevention intervention publications were analysed with 2015 (n = 6) the peak publishing year. Over half of the study designs were pre-post (n = 15). Intervention duration ranged from 4 hours to 11 years. Ten studies described either behaviour change theory or formative evaluation to inform design. Thirteen studies targeted interventions at a population level, seven at a group level and two at individual level. Studies identified a range of prevention strategies, categorised as behavioural (n = 9) (e.g., swimming lessons), socio-ecological (n = 8) (e.g., mandatory personal flotation devices) and mixed (n = 5) (e.g., awareness campaign and barriers to prevent access to water). A range of outcomes were described including changes in awareness, water safety knowledge, attitudes, water safety behaviours and skills, environmental, policy and regulation changes and drowning rates. Findings indicate a small but important increase in the evaluation and publication of effective interventions to prevent adult drowning. The complexity of the issues surrounding drowning requires multi-strategy and context -specific adult focused prevention interventions. Contemporary evidence that identifies effective interventions that contribute to prevention efforts is an essential first step in addressing the challenge.

## Introduction

Drowning is a global public health challenge. In 2019, more than 230 000 people drowned, with the majority (90%) occurring in low-income and middle-income countries (LMICs) [1, 2]. Close to two-thirds of all reported drowning deaths involved those aged 15 or older [1]. Both fatal and non-fatal drowning have significant impacts, including loss of household income and support, family breakdown and increased burden of care for survivors [3–6].

Drowning prevention in adults is a complex, multifaceted public health issue [7, 8]. Some risk factors (age, male gender, ethnicity, low socioeconomic status, and the use of alcohol) [9, 10] are consistent globally. However, others, for example weather, boat carrying capacity and watercraft design are specific to local social and cultural environments [11, 12].

Adult risk factors can be categorised as either behavioural (e.g., alcohol use, swimming alone and wearing a lifejacket) or socio-ecological factors (for example, water safety literacy, unsafe watercraft and unprotected infrastructure and equipment) [8, 13–15]. Globally, male drowning risk is high, with twice the overall mortality rate of females [16]. This disparity is primarily due to greater exposure to water and engagement in practices and behaviours which may place them at higher risk [8, 17, 18]. In high-income countries (HICs), males participate more frequently in recreational swimming, boating, and fishing [14, 19]. Risk is exacerbated by specific behaviours such as swimming alone, not wearing a personal floatation device (PFD) when boating or rock fishing, and alcohol consumption [8, 13, 14, 20, 21]. In contrast, in LMICs, males are exposed daily to natural waterbodies as a source of livelihood, using water transport to and from work, including in vessels that are often overcrowded and poorly maintained [12, 22, 23]. Age is also a significant factor in drowning risk. For example, young people (particularly males aged over 16 years) in HICs are at greater risk of drowning, attributed to risk-taking behaviours, peer pressure, poor aquatic competency and alcohol use [8, 21, 24, 25]. In contrast, for older adults (65 years and over) in HICs, risks relate to bathing, falls into water, poor health, alcohol consumption, medication use and lack of safety preparation around natural water bodies or during recreational activities [26, 27].

In 2016, the authors published a systematic review of adult drowning prevention interventions [8]. Only six studies met the inclusion criteria, briefly, three studies reported on interventions using educational strategies, one reported an environmental change intervention, and two studies reported legislative change interventions – all studies were located in HICs [8]. Studies were of mixed design and quality, with limited reference to theory or formative research. The 2016 review called for more robust studies underpinned by evidence-informed health promotion approaches [8]. More recently, the literature has seen an increase in the publication of research focusing on drowning prevention [28] supported by the impetus from the 2021 United Nations (UN) Global Charter for Drowning Prevention [29] which called for strengthened multi-strategy, multisectoral actions in drowning prevention around the globe. This updated systematic review aimed to assess the contemporary evidence base for adult -focused, public health interventions that address fatal and non-fatal drowning.

## Methods

The review replicated the steps for the previous review outlined by Leavy, Crawford [8] and Crawford, Leavy [30], in line with the Preferred Reporting Items for Systematic Review and Meta-Analyses (PRISMA) guidelines [31].

### Study Setting and Participants

The study setting and participants matched the description provided in the previous adult review [8]. Briefly, we analysed public health, primary studies, focusing on drowning interventions designed to prevent adult fatal or non-fatal drowning. For the purposes of the review, adults were defined as individuals over the age of 18. Eighteen years of age has been designated as the age at which childhood ends by the UNICEF Convention on the Rights of the Child [28]. Interventions aimed at both children/young people and adults were included where there was clear reporting on the impact of the intervention on adults.

### Criteria for Inclusion

Included articles met the following criteria: peer-reviewed, published in English between 2011 and 2021 that described and evaluated adult drowning primary prevention interventions targeting an individual, and/or group and/or population level. We included experimental and observational studies, randomised and non-randomised controlled trials, cohort studies, case-control studies, cross-sectional studies, retrospective analysis, and qualitative studies that evaluated primary adult drowning prevention interventions/strategies [8, 30] See Table 1. As established by the previous review, primary prevention strategies were those that eliminate or reduce causes of poor health, and/or promote protective factors. Secondary and tertiary intervention/s (those that include early detection, delaying complications, management, and rehabilitation, which occur after the possibility of prevention e.g., resuscitation) and reviews were excluded [8, 30].

**Table 1:** Inclusion and Exclusion Criteria

Inclusion Criteria	Exclusion Criteria
Published between 2011 and 2021	Children are the primary target group
Primary prevention intervention	Clinical trials
Must have an evaluation of the intervention	Therapy trials
English language	Medical interventions
Peer-reviewed publications only	Studies focusing on risk factors
≥ 1 Outcome measure assessed in this review must be addressed	Studies focusing on medical conditions
	Formative evaluation of intervention process and protocol studies
	Technical testing and evaluation of technology used in strategies/interventions

### Search Strategy

The research team searched 15 databases: PubMed; JSTOR; CINAHL; EMBASE; ERIC; ProQuest; PsycINFO; ScienceDirect; Scopus; Global Health; Web of Science; Current Contents; Wiley Online Library; Medline; and Sport Discus. Keywords and MESH terms were:

1. drown* adjacent to (prevent* or safety) within 3 words [MESH: Drowning/ pc [Prevention & Control].
2. interven* OR evaluat* OR “best practice” OR “good practice” OR “best practise” OR “good practise” OR “health promot*” OR “public health” OR polic* OR program* OR research OR prevent* OR education OR curriculum [MESH: Health Education/ and Preventive Health Services/].

#### Outcome Measures

Outcome measures included: drowning rates; water safety behaviour changes, or changes in behavioural intention or drowning awareness, knowledge, attitudes, water safety policy and legislation; changes to environment; and water safety skills.

### Selection of Articles, Screening and Quality Appraisal

**Fig. 1:**
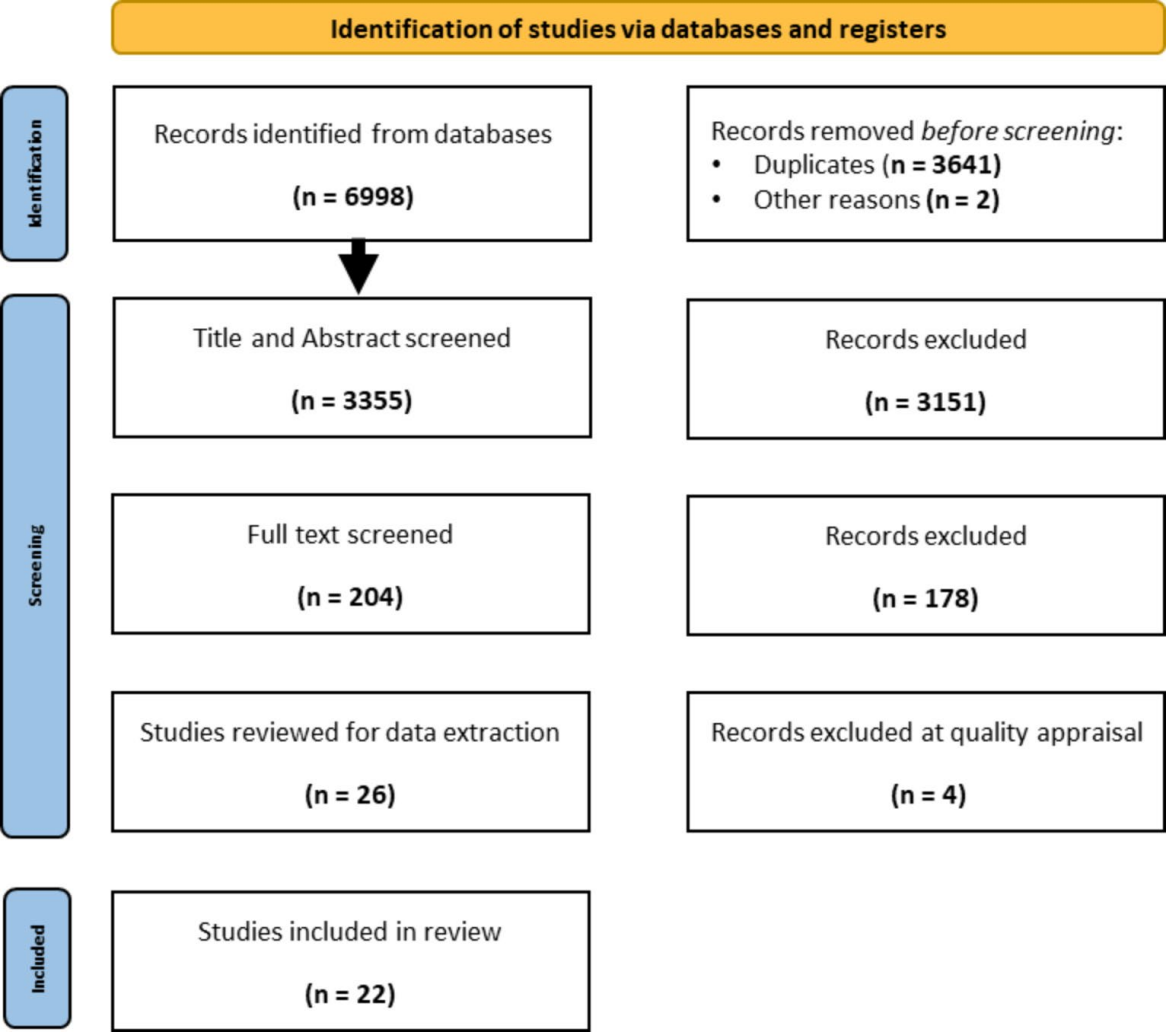
Systematics Review Flow Chart following PRISMA 2020 flow diagram for new systematic reviews template [31]

Endnote X9 [32] and Rayyan.AI [33] supported the selection and screening process [34]. Duplicates were removed, and two research assistants (AV) and (TG) screened article titles and abstracts for relevance against the inclusion criteria using-Rayyan.AI [33], a tool for screening and record-keeping to facilitate researcher collaboration. Excluded publications were scanned, and one research assistant (TG) randomly cross-checked 10% to identify any selection anomalies. The full-text articles from relevant studies that met the inclusion criteria were retrieved and reviewed by three reviewers (AV, JL & GC) to confirm eligibility for inclusion (see Fig. 1). Reviewer discussion resolved inconsistencies. The reference lists of all included articles were hand-searched for any relevant studies that had not been previously retrieved.

Twenty-six studies were quality appraised by two reviewers (JL and ND), using a purposively tailored quality appraisal checklist adapted from the MetaQAT framework [35]. The tool consists of four domains: relevancy; reliability; validity; and applicability [35]. Appraisers allocated a score to each of the nine criteria in the domains, whereby: Met the criteria = 2, Not sure/Unclear = 1; Did not meet the criteria = 0. A maximum score of 18 was allocated. Studies were then categorised based on the overall score, ≤ 9 = low quality, 10–14 = medium quality; and 15–18 = high quality. After the quality appraisal, four (n = 4) studies were reviewed independently by GC and deemed unsuitable for inclusion. A final sample of n = 22 studies were included in the revie.

### Data Extraction, Analysis and Synthesis

Data extraction replicated the procedure from the previous review. Briefly, descriptive and outcome data for all included articles were identified and recorded using a standardised data extraction form by two reviewers (AV, ND) and checked by a third reviewer (CG). Full-text articles were read and annotated to extract information under each of the following headings: author (year) & aims and objective; location & sample (n); recruitment & response rate; intervention level (individual, group, population), strategy type (behavioural defined as actions that individuals take concerning drowning prevention [36]; or socio-ecological defined as part of the social, physical and policy environment [37]), activity type (e.g. education, environmental, regulatory); duration; behaviour theory (BT) and formative research (FR); evaluation design; measures; ethical approval; and impacts/outcomes. Results were reviewed by the authors (JEL, GC, CG) for consistency.

Basic reporting of “pooled” data on age, gender and key findings are included. No meta-analyses were conducted due to the considerable heterogeneity across the studies.

## Results

### Setting, Duration and Evaluation Design

Twenty-two studies were included in the review (Table 2). The majority (n = 16) [38–53] were from HICs; six [54–59] were from LMICs. Australia (n = 7) [38, 39, 43, 46, 48, 52, 53] and the US (n = 6) [40–42, 47, 49, 51] were the two most represented geographical locations, representing over half of included studies.

**Table 2:** Review of drowning prevention interventions 2011–2021

Author Year Aim Location	Sample (n) Recruitment Response Rate	Intervention Level Strategy Type Activity Duration Use of theory (BT) Formative Research (FR)	Evaluation Design Measures Ethical Approval	Impacts/Outcomes
**Author**: Artiga, Limbo, Maningo, and Mamolo (2020) **Year**: 2020 **Aim**: To determine whether “Langoy sa Kaluwasan-Learn to Swim” extension project improved swimming skills of emergency response personnel. **Location**: Leyte, Philippines	**Sample**: Barangay emergency response personnel (n = 13). *Males*: 11 *Females*: 2 *Age*: 18–57 years **Recruitment**: N/R **Response Rate**: N/R	**Intervention Level**: Group **Strategy Type**: Behavioural **Activity**: Educational *Lecture, learn to swim program* **Duration**: 5 weeks **BT**: N/R **FR**: N/R	**Design**: Pre-post study **Measures**: Swimming skills. **Ethical approval**: N/R	**Water safety skills** Swimming skills significantly higher after intervention (< 0.005).
**Author**: Bugeja, Cassell, Brodie, and Walter (2014) **Year**: 2014 **Aim**: To investigate if mandatory PFD use reduces drowning deaths among recreational boaters. **Location**: Victoria, Australia	**Sample**: Recreational boaters (deceased) (pre n = 59, post n = 16). *Males*: 74 *Female*: 1 *Age*: 0–60 + years **Recruitment**: Coronial data **Response Rate**: N/A	**Intervention Level**: Population **Strategy Type**: Socio-ecological **Activity**: Regulatory *Introduction of mandatory Personal Flotation Devices and Other Safety Equipment Regulation 2005* **Duration**: 11 years **BT**: N/R **FR**: N/R	**Design**: Pre-post study **Measures**: Deaths pre-regulations to deaths post-regulations, use of LJ. **Ethical approval**: Yes	**Drowning rates** 16 drownings post-regulation compared to 59 pre-regulation. **Water safety behaviour** PFD wear increased from 22% (pre-regulation) to 63% (post-regulation).
**Author**: Cassell and Newstead (2014) **Year**: 2015 **Aim**: To evaluate the impact of the 2005 Victorian PFD regulations on PFD use by boaters in small power recreational vessels. **Location**: Victoria, Australia	**Sample**: Small vessel occupants in 2005 (n = 1,196) and 2007 (n = 1,062). Large vessel occupants in 2005 (n = 1,486) and 2007 (n = 1,285). *Males*: 3,889 *Females*: 1,114 *Age*: 0–60 + years **Recruitment**: Observation **Response Rate**: N/A	**Intervention Level**: Population **Strategy Type**: Socio-ecological **Activity**: Regulatory *Introduction of 2005 Victorian mandatory PFD regulations* **Duration**: 21–25 months **BT**: N/R **FR**: N/R	**Design**: Pre-post study **Measures**: PFD usage and type worn, vessel type, boater estimated age and sex. **Ethical approval**: Yes	**Water safety behaviour** Small power vessels PFD use increased from 22% in 2005 to 63% in 2007. Highest increase for small vessels with 60 + years at 33.5-fold. No significant increase in PFD use in large boat vessel users (12% in 2005 to 13% in 2007).
**Author**: Chung, Quan, Bennett, Kernic, and Ebel (2014) **Year**: 2014 **Aim**: To assess LJ use among boaters in Washington State and examine relationship between boating laws and LJ use. **Location**: Washington State, USA	**Sample**: Boaters (n = 5,157). *Males*: 3,222 *Females*: 1,828 *Age*: 0–65+ **Recruitment**: Observation **Response Rate**: N/A	**Intervention Level**: Population **Strategy Type**: Socio-ecological **Activity**: Regulatory *Laws and regulations associated with LJ use.* **Duration**: 1 month **BT**: N/R **FR**: N/R	**Design**: Observational study **Measures**: LJ use, vessel type, estimated boater age and sex. **Ethical approval**: Yes	**Water safety behaviour** 30.7% of all persons wore LJ. No significant difference in use by sex. LJ use highest among groups required by state law (96.8% of personal watercraft users, RR 3.7), 95.3% people being towed (water-skiers, RR 2.9) and children 0–12 years (81.7%, RR 4.2).
**Author**: Davoudi-Kiakalayeh, Mohammadi, and Yousefzadeh-Chabok (2012) **Year**: 2012 **Aim**: To describe effectiveness of a community-based drowning prevention intervention. **Location**: Guilan and Mazandaran provinces, Iran.	**Sample**: Rural residents of Caspian Sea coastline. Drowning deaths in sample area between 03/2005–03/2009 (n = 381). *Males*: N/R *Females*: N/R *Age*: 0–85 + years **Recruitment**: Coronial data **Response Rate**: N/R	**Intervention Level**: Population **Strategy Type**: Socio-ecological, Behavioural **Activity**: Educational, Environmental *Public health messaging in local TV programs & radio, distributing pamphlets, posters & notices, informational programs for healthcare providers, first responders educating clients about drowning, modification of environment (water reservoirs)* **Duration**: 4 years **BT**: N/R **FR**: N/R	**Design**: Pre-post study **Measures**: Deaths pre-intervention to deaths post-intervention **Ethical approval**: Yes	**Drowning rates** Fatal drowning incident rate fell from 4.5 per 100,000 at baseline to 3.6 per 100,000 at end of project.
**Author**: Davoudi-Kiakalayeh, Mohammadi, Yousefzade-Chabok, and Jansson (2013) **Year**: 2013 **Aim**: To evaluate a drowning prevention intervention package. **Location**: Guilan and Mazandaran provinces, Iran.	**Sample**: Residents and tourists in residential, coastal areas. Drowning deaths in sample area between 03/2005–03/2009 (n = 1,294). *Males*: N/R *Females*: N/R *Age*: 0–65 + years **Recruitment**: Coronial data **Response Rate**: N/A	**Intervention Level**: Population **Strategy Type**: Socio-ecological, Behavioural **Activity**: Educational, Environmental *Public health messaging in local TV programs & radio, distributing pamphlets, posters & notices, informational programs for healthcare providers, first responders educating clients about drowning, modification of environment (water reservoirs)* **Duration**: 4 years **BT**: N/R **FR**: Small scale implementation of intervention in both areas for 5 months, assessed community feedback through focus group discussions	**Design**: Pre-post study **Measures**: Drownings pre-intervention to drownings post-intervention **Ethical approval**: Yes	**Drowning rates** Fatal drowning incident rate reduced from 4.24 per 100,000 residents at baseline to 3.16 per 100,000 residents at end. Risk of drowning decreased from pre-intervention (OR = 1.15, 95% CI: 0.66–2.01) to end of study (OR = 0.24, 95% CI: 0.15–0.37).
**Author**: Girasek (2019) **Year**: 2019 **Aim**: To examine if potential legal and financial consequences have an impact on water-entry. **Location**: Virigina and Maryland, United States of America	**Sample**: Visitors to Potomac River Gorge. *Males*: N/R *Females*: N/R *Age*: N/R **Recruitment**: Observation **Response Rate**: N/A	**Intervention Level**: Population **Strategy Type**: Socio-ecological **Activity**: Environmental *New signage informing visitors of illegality of entering water and associated fine* **Duration**: 6 weeks **BT**: General Deterrence Theory **FR**: N/R	**Design**: Pre-post study **Measures**: People in water, degree of water entry (wading, above shoulders, etc.). **Ethical approval**: Yes	**Water safety behaviour** Experimental sign visible reduced odds of image showing someone in water by 63%. Sign condition (0.37 95% CI 0.14–0.99, p = 0.049) significantly associated with water entry.
**Author**: Mangione and Chow (2014) **Year**: 2014 **Aim**: To compare and evaluate the wear rates of LJ between two approaches; marketing campaign and mandatory regulation. **Location**: Central California, Delta Region and Mississippi, United States of America	**Sample**: Boating sites and lakes. *Educational campaign - “Wear it California”*: boaters (n = 49,951) on boats (n = 15,370) *Mandatory LJ regulations*: boaters (n = 25,166) on boats (n = 9,023) *Males*: 59,675 adults 2009–2011 *Females*: 31,404 adults 2009–2011 *Age*: 0–18 + years **Recruitment**: Observation **Response Rate**: N/A	**Intervention Level**: Population **Strategy Type**: Socio-ecological, Behavioural **Activity**: Educational, Regulatory *Mass media promotions, radio advertising, marina events, signing of pledge cards, celebrity appearances, campaign boat staffed with ambassadors, LJ regulations.* **Duration**: 6 years **BT**: N/R **FR**: N/R	**Design**: Pre-post study **Measures**: LJ use, boat type/size, boating activity **Ethical approval**: N/R	**Water safety behaviour** *Educational campaign*: Adult LJ wear increased from 8.5% pre-intervention to 10.5% post-campaign. *Mandatory LJ regulations*: Adult LJ wear increased from 13.7% pre-intervention to 68.1% post-intervention.
**Author**: Matthews, Andronaco, and Adams (2014) **Year**: 2012 **Aim**: To investigate to what extent warning signs on approach to beaches add to existing beachgoer knowledge. **Location**: Victoria, Australia	**Sample**: Beaches (n = 4), adult beachgoers (n = 472) *Males*: N/R *Females*: N/R *Age*: 18 + years **Recruitment**: Convenience sampling **Response Rate**: 89.9%	**Intervention Level**: Population **Strategy Type**: Socio-ecological **Activity**: Environmental *Hazards signage.* **Duration**: 3 months **BT**: Communication-Human Information Processing (C-HIP) model **FR**: N/R	**Design**: Cross-sectional study **Measures**: Hazard identification, signage & danger awareness **Ethical approval**: Yes	**Water safety knowledge** Less than half (45.0%) of respondents noticed any signage. 22.3% of respondents who passed signage gave currents/rips as first hazard compared to 43% (p < 0.001) of respondents who did not pass any signs.
**Author**: Moran (2016) **Year**: 2017 **Aim**: To determine effects of a decade-long safety intervention. **Location**: Auckland, New Zealand	**Sample**: Rock fishers at rock fishing sites 2016 (n = 165) 2015 (n = 413) *Males*: 2006: n = 229, 92% 2015: n = 378, 19% *Females*: 2006: n = 20, 8% 2015: n = 35, 9% *Age*: 15–65 + years **Recruitment**: Convenience sampling **Response Rate**: N/R	**Intervention Level**: Group **Strategy Type**: Behavioural **Activity**: Educational *Written material and verbal advice from field officers, safety messages via television, radio, newspaper and magazines.* **Duration**: 10 years **BT**: Protection Motivation Theory **FR**: Survey to identify fishers demographics and fishing safety knowledge, attitudes and behaviours.	**Design**: Cross-sectional study **Measures**: Water safety behaviours, perceptions of safety **Ethical approval**: N/R	**Water safety awareness** Increase in awareness of severity of drowning risk (70–91%) and vulnerability to drowning (50–72%) across the 10 years. **Water safety behaviour** Increase in self-reported often/always use of LJ from 4% in 2006 to 40% in 2015.
**Author**: Moran, Webber, and Stanley (2017) **Year**: 2017 **Aim**: To evaluate uptake of rescue information and emergency procedures from the *4Rs of Aquatic Rescue*. **Location**: Auckland, New Zealand	**Sample**: Parents/caregivers of children enrolled in water safety lessons (n = 476) at metropolitan Auckland swimming pools (n = 20) *Males*: 23% *Females*: 77% *Age*: 67% 30–44 years **Recruitment**: Convenience sampling **Response Rate**: 37%	**Intervention Level**: Group **Strategy Type**: Behavioural **Activity**: Educational *Water safety lessons (5 × 30 min)/, pamphlets, website page, downloadable resources, video link, social media campaign, newspaper releases* **Duration**: 4 months **BT**: N/R **FR**: N/R	**Design**: Pre-post study **Measures**: Estimated ability and willingness to rescue someone, understanding of rescue techniques **Ethical approval**: N/R	**Water safety knowledge** Significant increase in bystander rescue knowledge. **Water safety attitudes** Increase in self-estimated rescue ability and willing to perform a rescue, but not significant.
**Author**: Petrass and Blitvich (2018) **Year**: 2018 **Aim**: To measure the effect of an aquatic rescue intervention. **Location**: Victoria, Australia	**Sample**: First year Bachelor of Education (Physical Education) and Bachelor of Exercise and Sport Science students (n = 135) *Males*: 56.4% *Females*: 43.6% *Age*: 17–34 years, **Recruitment**: Convenience sample **Response Rate**: N/R	**Intervention Level**: Group **Strategy Type**: Behavioural **Activity**: Educational *1 h theory session (not compulsory) and 4 × 1 h compulsory practical sessions.* **Duration**: 4–5 h **BT**: Collaborative and social learning pedagogical approach **FR**: N/R	**Design**: Pre-post study **Measures**: Simulated rescue competency. **Ethical approval**: Yes	**Water safety knowledge** Rescue knowledge improved significantly (p < 0.001). **Water safety skills** Significant improvement in ability to perform a simulated rescue (p < 0.001).
**Author**: Quan et al. (2020) **Year**: 2021 **Aim**: To examine association between regulation of designated open water swim sites and open water drowning rates by state. **Location**: United States of America	**Sample**: Drowning deaths in open water swim sites of 30 states (n = 10,839) *Males*: N/R *Females*: N/R *Age*: 0–17, 18 + years **Recruitment**: Drowning data **Response Rate**: N/A	**Intervention Level**: Population **Strategy Type**: Socio-ecological **Activity**: Regulatory *Water quality monitoring, rescue/safety equipment, lifeguards, signage, reporting.* **Duration**: 6 years **BT**: N/R **FR**: N/R	**Design**: Retrospective analysis **Measures**: Presence of regulations relating to water safety, drowning deaths **Ethical approval**: N/R	**Drowning rates** 40% (12/30) of states had regulations pertaining open water swimming sites. Association between fewer regulations and higher drowning rates observed. States with only three (of five) regulations had drowning rates 2.56 times higher than states with all five regulations.
**Author**: Sansiritaweesook and Kanato (2015) **Year**: 2015 **Aim**: To develop an effective surveillance system to reduce drowning. **Location**: Ubon Ratchathani Province, Thailand	**Sample**: Drowning deaths in 26 villages *Males*: N/R *Females*: N/R *Age*: 3–70 years **Recruitment**: Drowning data **Response Rate**: N/A	**Intervention Level**: Population **Strategy Type**: Socio-ecological, Behavioural **Activity**: Educational, Environmental *Additional LJs made available, environmental improvements (fencing, signage), swimming lessons, drowning awareness campaign, resuscitation training* **Duration**: 15 months **BT**: N/R **FR**: 2-month situational analysis.	**Design**: Pre-post study **Measures**: Risk factor assessments through observation and interviews, drowning incidences. **Ethical approval**: Yes	**Environmental changes** Sites with LJs increased from 18.4% pre-intervention to 83.7% post-intervention. Physical environment security measures (fences, etc.) increased from 13.2–76.7% **Water safety skills** Child swimming ability rose from 38.5–52.0%. Proportion of rescue volunteers trained in lifesaving and resuscitation rose from 6–27.4%. Increase in village health volunteers trained in resuscitation (from 12.7–87.9%) **Drowning rates** Drowning incident rate decreased from 6.6/10,000 to 0.45/10,000 post-intervention. Incidence rate ratio of injuries was 23.32 (p = 0.002) times higher in comparison areas.
**Author**: Sansiritaweesook, Muangsom, Kanato, and Ratanasiri (2015) **Year**: 2015 **Aim**: To evaluate the effectiveness of a surveillance system for drowning prevention. **Location**: Ubon Ratchathani Province, Thailand	**Sample**: Household residents in intervention and control areas Intervention area: residents (n = 21,234), households (n = 5,667), communities (n = 26). Control area: residents (n = 14,805), households (n = 4,847), communities (n = 17). *Males*: N/R *Females*: N/R *Age*: N/R **Recruitment**: Drowning data **Response Rate**: N/A	**Intervention Level**: Population **Strategy Type**: Socio-ecological, Behavioural **Activity**: Educational, Environmental *Additional LJs made available, environmental improvements (fencing, signage), swimming lessons, drowning awareness campaign, resuscitation training* **Duration**: 7 months **BT**: N/R **FR**: N/R	**Design**: Pre-post study **Measures**: Risk factor assessments through observation and interviews, drowning incidences. **Ethical approval**: Yes	**Water safety behaviour** All risk factors reduced, including risky behaviour by child supervisor (33.5–12.4%) and risky behaviour by children (36.2–16.7%). **Water safety skills** Untrained rescue volunteer reduced from 87.4–12.1%. **Drowning rates** Drowning rate in intervention area reduced. Control area had a 5.6 times risk of injury (p < 0.05).
**Author**: Savage and Franklin (2015) **Year**: 2015 **Aim**: To evaluate AUSTSWIM’s training methods to ascertain most effective training for CALD community members. **Location**: New South Wales, Australia	**Sample**: CALD community members who participate in AUSTSWIM Teacher of Swimming and Water Safety training (n = 63). *Males*: 52% of survey *Females*: 48% of survey *Age*: 17–60 years **Recruitment**: Convenience sample **Response Rate**: N/R	**Intervention Level**: Group **Strategy Type**: Behavioural **Activity**: Educational *AUSTSWIM Teacher of Swimming and Water Safety course (2 full days, 16 h) delivered by AUSTSWIM public course (standard), course in Korean, course delivered using indigenous/CALD resources and mentoring* **Duration**: 7 months **BT**: N/R **FR**: N/R	**Design**: Cross-sectional study **Measures**: Swimming course completion **Ethical approval**: Yes	**Water safety skills** 52% of candidates had completed the course. This was highest amongst the indigenous/CALD course (83%), followed by the public course (57%) and Korean course (29%).
**Author**: D. C. Schwebel, H. N. Jones, E. Holder, and F. Marciani (2011) **Year**: 2011 **Aim**: To investigate if simulated drowning lifeguard audits improve lifeguard surveillance and reduce swimmer risk-taking behaviour at public pools. **Location**: Alabama, United States of America	**Sample**: American Red Cross-trained lifeguards across 14 swimming pools. *Males*: 50% *Females*: 50% *Age*: N/R **Recruitment**: Purposive sample **Response Rate**: N/R	**Intervention Level**: Group **Strategy Type**: Behavioural **Activity**: Regulatory *Audit of lifeguard and swimmer behaviour* **Duration**: 35 days **BT**: Health Belief Model **FR**: N/R	**Design**: Pre-post study **Measures**: Lifeguards’ looking behaviour, lifeguard warnings and swimmer risky behaviours. **Ethical approval**: Yes	**Water safety behaviour** Statistically significant increase in lifeguard looking behaviours at one-month follow up (p < 0.01). Non-significant increase in lifeguard warnings. Swimmer risky behaviours decreased post-intervention (p < 0.01).
**Author**: R. Scurati, G. Michielon, G. Signorini, and P. L. Invernizzi (2019) **Year**: 2019 **Aim**: To assess the benefits of video feedback via mobile device in learn-to-swim programs. **Location**: Italy	**Sample**: Sports science students (n = 16) *Males*: 8 *Females*: 8 *Age*: 20.6 ± 0.5 years **Recruitment**: Convenience sample **Response Rate**: N/R	**Intervention Level**: Individual **Strategy Type**: Behavioural **Activity**: Educational *Learn to swim program for breaststroke with augmented feedback through mobile devices (MDS) compared to traditional instructed gestured and verbal feedback control group (C).* **Duration**: 8 weeks **BT**: N/R **FR**: N/R	**Design**: Pre-post study. **Measures**: Swimming skills. **Ethical approval**: Yes	**Water safety skills** C and MDS methods both improved in breaststroke skills with no significant differences.
**Author**: Stempski et al. (2015) **Year**: 2015 **Aim**: To examine the impacts and feasibility of community partnership to change policy and systems to enhance access to safe swimming. **Location**: Washington State, United States of America	**Sample**: Community partners (n = 14). *Males*: N/R *Females*: N/R *Age*: N/R **Recruitment**: Purposive sample **Response Rate**: 100%	**Intervention Level**: Population **Strategy Type**: Socio-ecological **Activity**: Regulatory *Policy and system changes.* **Duration**: 18 months **BT**: Socio-ecological model **FR**: Partnerships with community organisations, focus groups with community members.	**Design**: Pre-post study **Measures**: Policy and system changes. **Ethical approval**: Yes	**Water safety policy and regulation** 58/73 identified policy changes made by one or more partners by end of grant. Policies increased in areas of screening, referrals and scholarships.
**Author**: Torlaković and Kebat (2015) **Year**: 2015 **Aim**: To analyse the efficiency of an individual training programme for non-swimmer adult women with fear of swimming. **Location**: Sarajevo. Bosnia	**Sample**: Female non-swimmers (n = 20) *Males*: N/A *Females*: 20 *Age*: 26–59 years **Recruitment**: N/R **Response Rate**: N/R	**Intervention Level**: Individual **Strategy Type**: Behavioural **Activity**: Educational *Individual swimming training (20 × 60 min sessions)* **Duration**: N/R **BT**: N/R **FR**: N/R	**Design**: Pre-post study **Measures**: Swimming skills. **Ethical approval**: N/R	**Water safety skills** Significant increase in swimming distance, swimming knowledge, and swimming technique (p < 0.001).
**Author**: Warton and Brander (2017) **Year**: 2017 **Aim**: To evaluate the educational effectiveness of *Bondi Rescue* in communicating information on beach safety and hazards. **Location**: Global	**Sample**: Viewers of *Bondi Rescue* worldwide (n = 1,852) 36.8% Australia, 34.9% UK and Ireland, 14.4% rest of Europe, 9.8% North America, 1.9% Asia, 1.1% Africa, 0.8% rest of Pacific, 0.3% South America, 0.2% Middle East *Males*: 14% *Females*: 86% *Age*: N/R **Recruitment**: Convenience sample **Response Rate**: N/R	**Intervention Level**: Population **Strategy Type**: Behavioural **Activity**: Educational *Bondi Rescue television program* **Duration**: 12 years lifeguard records, 68 days viewer survey **BT**: N/R **FR**: N/R	**Design**: Cross-sectional survey **Measures**: Beach hazards knowledge, beach safety and education knowledge. **Ethical approval**: Yes	**Water safety knowledge** Respondents felt that watching *Bondi Rescue* improved awareness rip current hazard (60%) and awareness of beach safety and hazard (96%). 17% felt they had learnt rescue skills.
**Author**: Willcox-Pidgeon, Franklin, Devine, Leggat, and Scarr (2020) **Year**: 2020 **Aim**: To describe evaluation of swimming and water safety programs delivered to adult migrants. **Location**: New South Wales, Australia	**Sample**: Adults from migrant groups currently undertaking or recently completed free adult swimming and water safety (n = 35). *Males*: 0 *Females*: 35 *Age*: ≥18 years **Recruitment**: Nonprobability purposive sampling **Response Rate**: N/R	**Intervention Level**: Group **Strategy Type**: Behavioural **Activity**: Educational *Swimming lessons* **Duration**: N/R **BT**: Theory of Planned Behaviour and Health Belief Model. **FR**: N/R	**Design**: Qualitative study **Measures**: Swimming program outcomes **Ethical approval**: Yes	**Water safety skills** Participants described sense of achievement in developing swimming skills, increased confidence in water, and awareness of water safety.

The intervention study designs included pre-post studies (n = 15) [39, 41, 42, 45, 46, 49–51, 54, 57–59] [38, 55, 56], cross-sectional studies (n = 4) [43, 44, 48, 52], an observational study [40], a retrospective analysis [47] and a qualitative study [53].

**Fig. 2:**
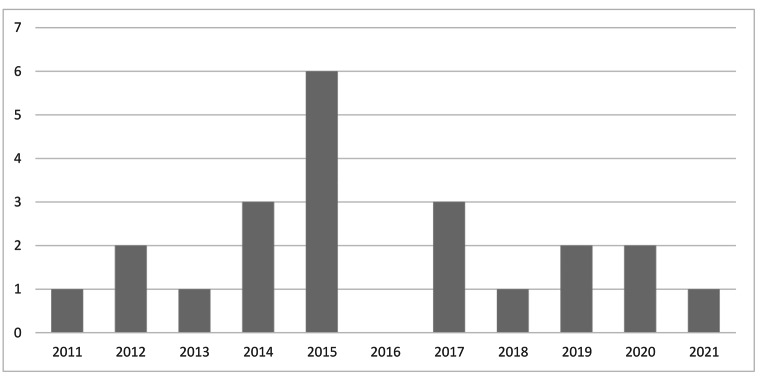
Yearly Trend of Adult Drowning Prevention Intervention Studies

There were two female-gender specific studies [53, 59]. The duration of drowning interventions ranged from 4 to 5 hours [46] to 11 years [38] while sample sizes ranged from 13 [54] to > 75 000 [42]. Ethical approval was reported for 15 studies [38–41, 43, 46, 48, 50–53, 55–58]. Yearly trends in drowning prevention intervention publications were analysed (Fig. 2). demonstrating one peak publishing year (2015).

### Drowning Prevention Strategies

Two studies [50, 59] presented interventions targeting individuals while seven studies presented interventions targeting groups [44–46, 48, 49, 54]. All but one [49] of these studies utilised behavioural strategies (Table 3). Behavioural activities were reported as educational, involving swimming lessons [46, 48, 50, 53, 54, 59] or educational materials (i.e., safety messages via field officers) [44, 45]. The study by Schwebel et al. (2011) used a regulatory audit to assess lifeguard behaviour.

The remaining thirteen studies [38–43, 47, 51, 52, 55–58] reported on interventions targeting a population, and included the use of behavioural approaches [52], socio-ecological approaches [38–41, 43, 47, 51] or a mix of strategy types [42, 55–58]. For example, the population-level behavioural intervention by Warton and Brander (2017) evaluated the effectiveness of the television show *Bondi Rescue* on water safety knowledge. The four studies involving both behavioural and socio-ecological interventions used a combination of educational material (i.e., drowning awareness campaigns) and changes in environment (i.e., barriers to water, increased lifejackets available) [55–58], with one study [42] comparing differences between an educational or regulatory intervention. Of the studies applying a socio-ecological approach, five studies were regulatory, involving the introduction of mandatory personal floatation devices (PFDs) [38–40] or other regulations [47, 51]. Two studies were environmental, involving signage [41, 43].

**Table 3:** Overview of studies by intervention level, strategy type and activity

	Individual	Group	Population	*Total*
Behavioural	2	7	1	10
*Educational*	*1*	*6*	*1*	
*Environmental*	*-*	*-*	*-*	
*Regulatory*	*1*	*1*	*-*	
**Socio-ecological**	**-**	**-**	**7**	**8**
*Educational*	-	-	-	
*Environmental*	-	*-*	*2*	
*Regulatory*	-	*-*	*5*	
**Mixed**	**-**	**-**	**5**	**5**
*Educational & Environmental*	-	-	*4*	
*Educational & Regulatory*	-	-	*1*	
*Regulatory*	-	-	-	
**Total**	**2**	**7**	**13**	**22**

### Behavioural Theory and Formative Research

Six studies (27%) reported the use of behavioural theory [41, 43, 44, 46, 49, 51, 53]. Two studies used the Health Belief Model [60], one applied it to swimming classes [53] and the other to lifeguard surveillance [49]. Other theories included the Socio-Ecological Model [51], Protection Motivation Theory [44], Theory of Planned Behaviour [53], General Deterrence Theory [41], Communication-Human Information Processing Model [43] and general considerations of pedagogical approaches [46].

Four studies (18%) described using some form of formative research to inform the intervention design and delivery and profile the target audience. Methods included a cross-sectional survey [44] and focus group discussions [51] with community members. Sansiritaweesook et al. (2015) reported using a two-month situational analysis before intervention development. Davoudi-Kiakalayeh et al. (2013) scaled their intervention based on a previous pilot intervention in the community.

### Intervention Outcomes

Outcomes described included: changes in awareness, water safety knowledge, attitudes, water safety behaviours and skills, together with environmental, policy and regulation changes and drowning rates. All but two studies reported some intervention effect [43, 50]. Scurati et al. (2019) assessed two different forms of feedback (traditional or through mobile devices) in a breaststroke swim program but found no significant differences. A cross-sectional survey by Matthews et al. (2014) asked beachgoers about hazard signage but found better knowledge of hazards on beaches without signs. Savage and Franklin (2015) found culturally and linguistically diverse (CaLD) participants more likely to complete a swimming and water safety course when using specific Indigenous/ CaLD resources than with the standard course, but less likely to complete the Korean course compared to the standard course. Six studies measured changes across multiple outcome categories [38, 44–46, 57, 58].

Eight studies (36%) measured water safety behavioural changes, with five reporting increases in the use of PFDs or lifejackets [38–40, 42, 44]. Other reported behavioural changes included reduced water entry [41], reduced incidence of risky swimming behaviour [58], and improved lifeguard-looking behaviour [49]. Eight studies (36%) measured changes in water safety skills, with six describing improvements in swimming ability [48, 50, 53, 54, 57, 59]. The study by Petrass et al. (2018) measured simulated rescue competency, and the two studies by Sansiritaweesook and colleagues (2015a, 2015b) reported increases in the proportion of volunteer rescue personnel with resuscitation training [57, 58].

Six studies (27%) assessed changes in drowning rates [38, 47, 55–58]. Four studies assessed knowledge, with three relating to rescue knowledge [45, 46, 52], and the study by Matthews et al. (2014) reporting no change in hazard knowledge. Other outcomes included environmental changes (increased lifejackets and rescue boats) [58], improved attitudes towards rescue [45], and increased number of water safety policies and regulations [51].

### Self-reported Study Limitations

Four studies did not report any limitations [43, 46, 52, 59]. Self-reported limitations relating to recruitment and participants included: a low response rate [48] and a small sample size [48–50, 53], convenience sampling [44, 45, 53], and English-only survey instruments [45, 48]. Limitations relating to study design included: short-term intervention [54], no control or comparison group [38, 55, 56], self-reported behaviour [44], selection bias [47], cross-contamination between groups [58], and lack of generalisability to other settings/groups [57]. Studies using observational data noted the potential for observational bias or misclassification of data [39, 40, 42, 61]. Girasek (2019) stated that the introduction of a camera may have influenced behaviour and enhanced the effectiveness of the intervention.

## Discussion

This study updates a previous systematic review undertaken in 2016 [8]. This review noted a three-fold increase in studies (from six to twenty-two articles), published between 2011 and 2021, that reported on public health interventions for adults. Of note, a recent bibliometric analysis found 39% of drowning research publications were from Australia and the US [28]. We found similar results with 37.5% of intervention studies from Australia and the US, and most (n = 16) from HICs. Interventions varied by intervention level, duration and design across the 22 studies. Nearly half of studies described the use of behavioural theory or formative research, and all but two interventions reported some intervention effect. Overall, the review found some positive shifts in the design and evaluation of adult focused drowning prevention interventions highlighting the vital contribution of research and evidence for interventions delivered across countries and communities globally.

Promisingly, and in line with contemporary health promotion and prevention approaches [62], more than half (n = 13) of the included studies in this review focused on population-level interventions using a socio-ecological or mixed strategy approach. In comparison, our previous review found a predominance of behavioural-only strategies emphasising education [8]. While behavioural interventions have merit, changes are often short-term. They may have limited impact on longer-term, population-level outcomes [63], such as fatal drowning events. In comparison, socio-ecological strategies often involve sustained interventions, such as regulatory or environmental changes, that are more likely to produce population-level outcomes [64]. Regulatory or environmental changes are more likely to be cost-effective long term [65] and translate to sustained behaviour change [66]. In this review, six studies assessed changes in drowning rates following either the introduction of regulatory PFD usage or environmental changes (e.g., increased barriers to water or availability of rescue equipment), with all demonstrating a considerable reduction in the number of drownings in the population sustained over time. The novel study by Girasek (2019) used signage and a considerable fine to achieve change. The study is an important reminder that multi-strategy prevention efforts targeting both the person at risk and structural considerations such as using regulatory options are more likely to be successful than programs relying on a single strategy. This finding is consistent with the broader literature on health promotion and prevention [62, 67]. By comparison, studies in this review focusing on behavioural-only strategies generally reported on short-term changes, such as knowledge and swimming skills, often amongst a small sample size and within a short time frame.

Socio-ecological strategies may also be more effective than behavioural-only campaigns for some behavioural measures [67]. For example, Mangione and Chow (2014) observed differences in lifejacket use between an educational lifejacket campaign and mandatory lifejacket regulations. Mandatory regulations increased lifejacket use by more than 50%, whilst the educational campaign increased lifejacket use by only 2% and was unsustained. While the authors could not accurately estimate the costs of implementing campaigns, they noted that the educational campaign required sizeable ‘new’ expenses. At the same time, mandatory regulation added little cost to existing activity [42]. Similarly, Chung et al. (2014) observed lifejacket use was considerably higher among boat users who were legally required to wear a lifejacket (96.8%) compared with boat users not wearing a lifejacket (21.1%). Comparatively, after a decade-long educational campaign in New Zealand, Moran et al. (2017) found only 40% of rock-fishers self-reported always/often wearing a lifejacket. A recent similar project in Victoria, Australia reported that their three-year educational campaign with rock fishers produced no change in lifejacket use [68]. The findings of the review highlight the availability, and regulation of lifejacket use as a critical and cost-effective approach to drowning prevention.

Those in public health have long advocated for the use of theory to plan, deliver, and evaluate interventions [69]. In this review, the use of behavioural theory and formative research was limited, similar to the findings of our previous reviews with adults [8] and children [7]. Studies used a range of theory with varying levels of application. In general theories were mentioned, but description of their application – to design and evaluation – was limited. Of note, one recent Australian qualitative study [53] described and mapped constructs from two theories, Health Belief Model [70] and the Theory of Planned Behaviour [71]to guide the focus group and individual interview question guide. This study provides an exemplar for future drowning prevention evaluation research. Similarly, Stempski et al. (2015) used the Socio-Ecological Model [72] to outline how policy and systems at a community level may help to improve the factors that influence swimming and water recreation. The use of formative research was limited in included studies, despite being a critical component of effective public health practice [73]. The use of theory has been highlighted as instructive to improve our understanding of the complexities of health behaviour and the environment which influences them [74].

Studies mostly reported positive outcomes from their interventions. Our previous review called for an increase in appropriate study designs, objective, valid and reliable measures and quality evaluations over a sufficient period [8]. The current review found the most frequently reported outcomes included water safety behaviour (mainly the use of PFDs) and water safety skills (predominately swimming skills). These outcomes were mainly evaluated through a pre-post study design, using observational data. While issues of observational bias and misclassification of data were reported, they may be more reliable than self-reporting of behaviour and skills [74]. However, several of these studies had relatively short timeframes of less than a few months [41, 45, 46, 49, 50, 54, 59] and/or were conducted with small samples [49, 50, 53, 54, 59], meaning it is unknown as to whether behaviour and skills were sustained over time [74], and likely to impact on both fatal and non-fatal drowning outcomes. Despite being published post the previous review, these studies do not incorporate previous recommendations. Promisingly, six studies measured changes in drowning rates, using a pre-post analysis to compare data across several years. All studies demonstrated a positive impact of interventions on drowning rates, though causation cannot be assumed. Only four studies used a cross-sectional design, measuring a mixture of knowledge, attitudes, skills and behaviour. Previous reviews have cautioned against reliance on self-report measures [7, 8, 24]. Overall, findings suggest incremental improvements in use of robust and standardised measures by researchers and practitioners, although improvements are warranted.

While almost 90% of drownings occur in LMICs [1, 2], the majority (n = 16) of articles included in this review were from HIC settings. Interventions in LMICs are delivered by a range of government and community-based agencies, that may not always have the capacity or resources for large-scale evaluation and knowledge translation [28, 75, 76]. Alternatively, knowledge translation may not be a priority or evaluation results may not be disseminated widely [77–79] or undertaken through other means, such as the grey literature [79, 80]. Of interest, a recent scoping review of the grey literature identified intersections for drowning prevention within global reports for occupational, environmental and urban health, refugee and migrant safety and disaster risk reduction, identifying potential partners and sectors to increase drowning prevention efforts [79] across a variety of settings. Additionally, while population approaches to drowning prevention are needed, there remains a role for targeted programs for potential at-risk groups, such as migrants or people from lower socioeconomic backgrounds [10, 53]. Three studies in this review focused on migrants [48, 53] or female non-swimmers [59]; however, the quality of design varied, sample sizes were small, and one study did not report an improvement [48]. Further evidence of approaches that work with different groups across different settings to address inequities in drownings is warranted.

### Strengths and Limitations

This study updates a previous review of drowning prevention interventions amongst adults, totalling 31 years of peer-reviewed literature. Strengths include searching fifteen databases, providing a broad scope, and following procedures from previously published systematic reviews [7, 8]. Additionally, use of the MetaQAT tool for quality appraisal is viewed as a strength as it is a public health specific tool. A teams-approach was adopted to finalise article selection, minimising potential errors. Several limitations are noted. Included articles were restricted to the English language, and grey literature was excluded. We acknowledge that non-English articles and the grey literature may yield valuable information on drowning prevention interventions. Community-based agencies are likely to deliver most interventions but may be less likely to publish in a peer-reviewed forum; therefore, their contributions may have been excluded. Despite these limitations, this review provides a contemporary overview of drowning prevention interventions for adults in low-, middle- and high-income countries.

## Conclusion

Drowning prevention in adults is a complex, multifaceted issue of public health significance but evidence for intervention development has been lacking This review updates the evidence base for adult-focused, public health interventions that address fatal and non-fatal drowning. Since our last review there has been a modest increase in the number of interventions published, together with some positive gains in design, delivery and evaluation over the past decade. Findings reinforce global calls for multi-level, multi-strategy approaches to intervention design, implementation and evaluation for maximum gains aligned with contemporary health promotion and prevention approaches.
